# Modified Method of T-tube Placement in Cases of Ruptured Choledochal Cyst Having Complete Loss of Anterior Wall

**DOI:** 10.4103/1319-3767.74452

**Published:** 2011

**Authors:** Ahmed Intezar, Rawat D. Jile, Anshuman Sharma, Anand Pandey, Ashish Wakhlu, Shiv N. Kureel

**Affiliations:** Department of Pediatric Surgery, CSM Medical University (Formerly King George’ Medical College), Lucknow – 226 003, India

**Keywords:** Biliary peritonitis, choledochal cyst, T-tube

## Abstract

Survival rates for infants and children who have choledochal cyst with or without spontaneous rupture have improved dramatically in the past decades. Despite excellent long-term survival for patients with choledochal cyst who undergo elective surgery, many significant complications can occur in the patients being operated in emergency for rupture of the cyst. Spontaneous rupture of the cyst is one such problem resulting in considerable morbidity and mortality in these patients. Majority of surgeons manage these cases with T-tube external drainage. The conventional methods of T-tube placement for long period has remained simple as described in choledochotomies where there is no deficit of the walls of common bile duct (CBD). The present technique has been designed specially for the cases of ruptured choledochal cyst, where the wall of the CBD gets necrosed leaving behind a long gap between the two ends. In these cases, placement of T-tube with conventional method is not possible because there is no wall to suture together, and make the CBD water tight again to prevent leakage of bile. We found only two patients of spontaneous rupture of choledochal cyst with a long gap between two ends of CBD because of necrosed anterior wall. In both of these patients, it was not possible to put T-tube with traditional method and one would have to opt for primary definitive repair despite poor general condition of patients.

The history of surgical treatment of choledochal cyst is remarkable, and more than 3000 cases have been reported in literature.[1] The majority of surgeons used to manage spontaneously ruptured choledochal cyst with T-tube external drainage,[1–3] and many researchers believe that it should be treated by primary definitive repair.[4,5] The method of placement of T-tube in the cases of spontaneously ruptured choledochal cyst for long time has remained the approximation of the edges of perforation site after putting T-tube into it.[6] But if perforation is big enough leaving behind only a small strip of posterior wall of the cyst [Figure 1], approximation of the two edges will be almost impossible after putting T-tube. At our institution, we have utilized “purse-string” sutures - a simple, modified technique of T-tube placement in cases of choledochal cyst rupture where traditional technique of T-tube placement failed.

**Figure 1:**
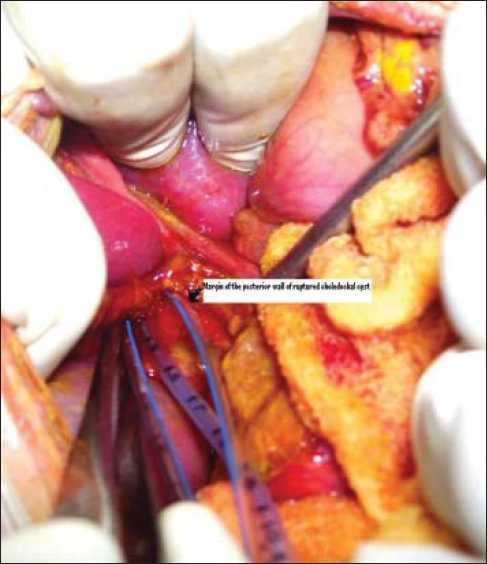
Intraoperative photograph showing ruptured choledochal cyst with a feeding tube inside upper end of common bile duct

## METHODS

A total of five patients of choledochal cyst with spontaneous rupture were admitted to our center in the last 10 years. Out of these five patients, only two patients (one 13 months/ female and another 2.5 years/male) had complete loss of about two-third of the circumference anteriorly, leading to a long gap between the two ends of the common bile duct (CBD). Both of these patients underwent the modified technique of T-tube placement while the other three patients underwent the traditional method of T-tube external biliary drainage. Broad spectrum antibiotic coverage and pulmonary physiotherapy were initiated. Intravenous fluid therapy with 10% dextrose and salt solution was started to maintain fluid, electrolyte and glucose balance. Vitamin K analogue were also administered before surgery.

## TECHNIQUE

**Figure 2:**
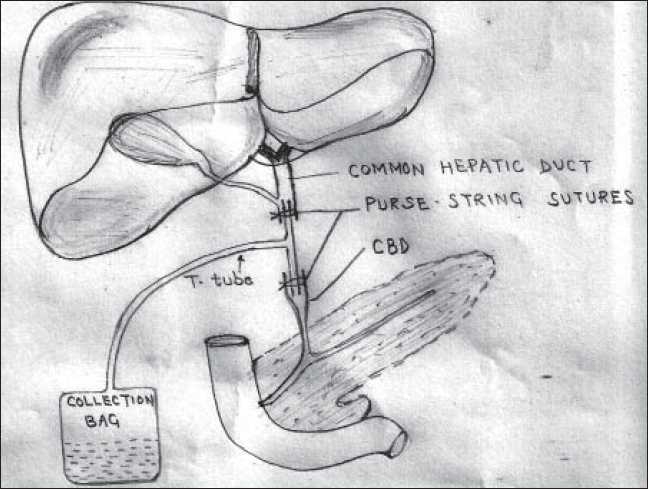
Diagrammatic representation of our T-tube placement technique

The modified technique employed in placement of T-tube is; putting “PURSE-STRING” sutures on both the ends of CBD after introduction of T-tube limbs into the ends of CBD, whereas in conventional method we used to approximate both the edges of the hole made spontaneously in anterior wall of choledochal cyst. This suture technique is well known and is used elsewhere, but never has been described in T-tube placement in cases of choledochal cyst with spontaneous rupture. After making the anatomy clear of the upper and lower end of the CBD, the purse-string sutures were applied with vicryl 3-0. Starting from the anterior wall, purse-string sutures were taken by taking equal amounts of tissues in each bite, taking care not to injure portal vein or hepatic artery, and finally, ends of sutures were tied to make CBD water tight [Figure 2]. One or two purse-string sutures were taken on each end of CBD.

**Figure 3:**
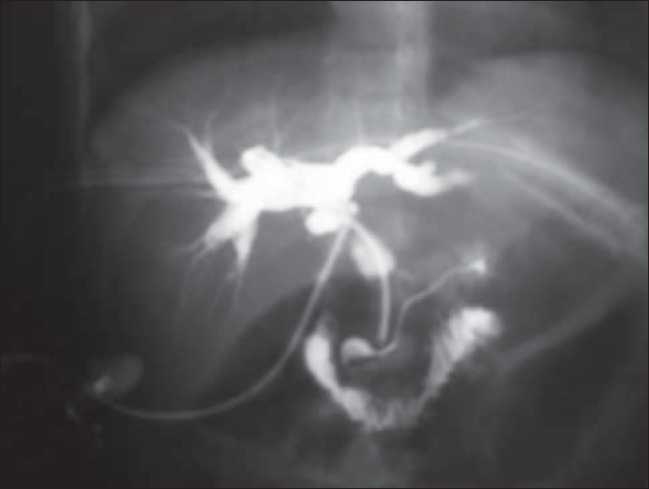
T-tube cholangiogram showing pancreatic duct, intrahepatic bile ducts, and duodenum

In this modified technique, we never cut-off a strip of the wall of the T-tube, as was done in the traditional method. We kept the limbs of T-tube small so they could not enter into hepatic duct or into the duodenum. An abdominal drain was put in all the patients in subhepatic space to drain out leaked bile from CBD, which did not occur in any of our patients. Long limb of T-tube was taken out through lateral wall of the abdomen and connected to a plastic bag. Abdominal drains were removed, if there was no output for 48 h. T-tube cholangiogram was ordered 10-14 days after operation Figure 3.

## DISCUSSION

Spontaneous rupture of choledochal cyst had been considered as rare and this complication has been found in 26 of 1,433 patients (1.8%) collected from Japanese literature, in 4 of 188 patients (2.1%) collected by surgical section of American Academy of Pediatrics and in 1 of 36 patients (2.8%) from an English series. However, it has been absent from other reports of large series of patients from Canada, Taiwan, and United States.[1] Since rupture can sometimes be the initial manifestation of the disease, it should be considered in the presence of bile-like fluid at the time of emergency laparotomy.[3]

Most reported cases have been managed with external drainage of the cyst followed by a second procedure to excise the cyst and reconstruct the biliary tract[1–3] as in our case, but many researchers recommend primary reconstructive surgery as the treatment of choice.[4,5] In developing countries, patients usually present late with much of septic load which do not allow for primary reconstructive surgery. Availability of the specialist concerned is also a limiting factor for primary reconstructive repair, especially in third world countries.

In most cases, there is only a small hole on the anterior wall of the cyst in cases of cyst rupture; but in a few cases, especially in those who present late, the whole of the anterior wall gets sloughed off after necrosis. In the cases where there is a small hole only in anterior we can place a T-tube with the conventional method i.e., approximating the edges of the hole after introducing T-tube into it. But in some cases where almost two-third of the cyst circumference is lost, with a long gap between two ends of the CBD, the traditional method fails and the only option left is primary definitive repair regardless of the general condition of the patient. In the technique used by us, one can put the T-tube even in cases having a complete loss of the anterior wall, and a large gap exists between two ends of CBD. This technique allows physiological flow of bile to start from the hepatic duct to the duodenum, along with external drainage. Abdominal drain output can reveal whether there is any bile leak or not. None of our patients had bile-leak, but as number of patients were limited in this study, larger studies with more number of patients are needed to evaluate it prospectively. T-tube cholangiogram after 10-14 days of operation will show biliary anatomy and flow of bile into duodenum; this is also the time the patient requires to recover from acute critical illness. T-tube external biliary drainage is just a bridging procedure between ruptured choledochal cyst and definitive repair, to buy time if general condition of the patient is low and if the patient is at a remote health care centre and an expert may not be available. As curative treatment for choledochal cyst is definitive reconstructive surgery, we had done excision of cyst along with Roux-en-Y hepaticojejunostomy in all the patients after six weeks of T-tube drainage. During excision of cyst, we had removed entire remnants of choledochal cyst which also include part of CBD where we had applied purse-string sutures during the previous surgery, so chances of long-term biliary fistula and stenochoria secondary to T-tube insertion are almost nil.

Recently, a few researchers have reported the role of percutaneous transhepatic cyst drainage in cases of perforated choledochal cyst.[7] The major limitations of this technique are availability of interventional radiology facilities and presence of biliary peritonitis in majority of patients with ruptured cyst having loss of major portion of its wall.

## CONCLUSION

Although it may not be an optimal method of T-tube placement, our method is successful in putting T-tube in cases having complete loss of the anterior wall hence minimizing the chances of postoperative leak of bile and buying the time for definitive repair, thereby subsequently reducing the morbidity and mortality in these patients.
